# Modeling the Influence of a Magnetomechanical Effect on the Permeability Tensor of a Tensductor Core

**DOI:** 10.3390/ma12244023

**Published:** 2019-12-04

**Authors:** Roman Szewczyk, Michał Nowicki, Anna Ostaszewska-Liżewska, Mika Malinen

**Affiliations:** 1 Warsaw University of Technology, Institute of Metrology and Biomedical Engineering, Boboli 8, 02-525 Warsaw, Poland; m.nowicki@mchtr.pw.edu.pl (M.N.); a.ostaszewska@mchtr.pw.edu.pl (A.O.-L.); 2 CSC-IT Center for Science, P.O. Box 405, FI-02101 Espoo, Finland; Mika.Malinen@csc.fi

**Keywords:** magnetoelastic effect, soft magnetic materials, amorphous alloys, principal stresses

## Abstract

This paper presents a new method of modeling the influence of mechanical stresses on a magnetic permeability tensor of soft magnetic materials. The proposed method utilizes the principal stresses concept to compensate the influence of shear stresses. As a result, the stress dependence of a magnetic permeability tensor may be assessed with only the knowledge about the influence of axial stresses on magnetic properties of isotropic material. The proposed method was used for a finite element method based model of a tensductor designed for measurements of tensile forces. Due to the fact that 2D stresses distribution occurs in a tensductor, simplification of both principal stresses and a magnetic permeability tensor rotation procedure was proposed. As a result, good agreement was reached between the results of modeling and the results of experimental tests. This result validates the possibility of utilization of the proposed modeling method for the design of magnetomechanical devices.

## 1. Introduction

Magnetoelastic phenomenon [1,2] is connected with the significant changes of magnetic permeability of magnetic materials subjected to the influence of mechanical stresses [3]. Known also as the Villari or inverse magnetostriction effect, it is one of the better known manifestations of wide range of magnetostriction-related phenomena [4,5]. This effect has great technical importance, such as the possibility of utilization of magnetoelastic effect in the development of robust mechanical stress and force sensors for industrial automation [6,7,8], biomedical engineering [9], and security [10]. On the other hand, an uncontrolled magnetoelastic effect may lead to degradation of magnetic properties of inductive cores made of magnetic materials accidentally subjected to mechanical stresses. In such a case, a magnetoelastic phenomenon may lead to a decrease of efficiency of switching mode power supplies [11] or a reduction of accuracy of the operation of fluxgate sensors [12].

In spite of the importance of the magnetoelastic effect for the development of devices with cores made of soft magnetic materials, knowledge about its physical background and mathematical model description is still intensively investigated [13,14]. Existing models are mostly focused on the influence of mechanical stresses parallel [15] or perpendicular [16] to the direction of magnetization of magnetic material. The complete description of the stress dependence of a magnetic permeability tensor seems to be still not presented in the literature.

The modeling concept presented in the paper is filling this gap. This method of modeling the stress dependence of a permeability tensor in two-dimensional systems may be utilized in a finite element method (FEM) modeling of magnetic devices with flat magnetic cores, or with cores which exhibit planar symmetry. An example of such a device is a tensductor [17], the tensile stress sensor utilizing changes of flux density of a ribbon made of amorphous alloy for measurements of tensile forces. Moreover, the same concept may be used for modeling the characteristics of pressductors [18] introduced by the Assea company, and now widely used for large compressive force measurements in industrial automation. In the case of these sensors, the measurement characteristic is not determined by the changes of dimensions of sensors under mechanical stresses, but by the changes of the material’s magnetic permeability tensor influenced by stress-induced magnetoelastic anisotropy. It should be highlighted that, until now, it was not possible to perform modeling of the output characteristics of such sensors with the finite element method, which significantly limited possibilities to optimize the sensor’s performance during the development process.

## 2. Modeling the Magnetoelastic Effect in 2D Systems 

Magnetoelastic characteristics of soft magnetic materials for the mechanical stresses applied in the same direction as magnetizing fields were widely presented in the literature [19]. Under the influence of axial tensile stresses *σ* in the elastic region, the permeability of soft magnetic material with a positive saturation magnetostriction constant *λ*_s_ increases, whereas for material with negative saturation magnetostriction the permeability decreases [20]. 

**Figure 1:**
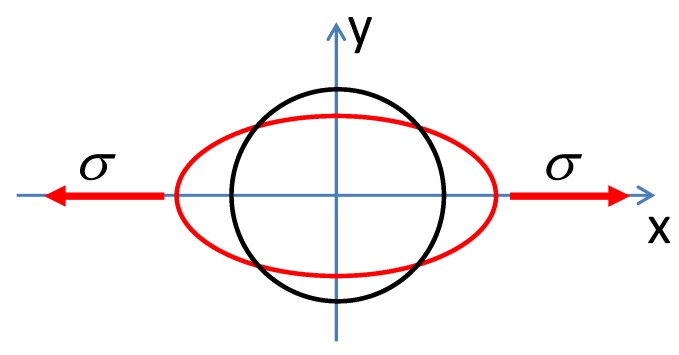
Permeability tensor *µ* in polar coordinate system: isotropic material (black line), magnetic materials with the positive saturation magnetostriction *λ_s_* subjected to tensile stresses *σ* in X direction (red line).

In the case of mechanical stresses $\sigma_{⊥}$ perpendicular to magnetization direction [21], it was confirmed [22] that effective stresses *σ_e_* influencing the magnetic properties may be assessed from the equation:

(1) $$\sigma_{e}=-\nu \cdot \sigma_{⊥},$$

where $\sigma_{⊥}$ is the value of stress perpendicular to the magnetizing axis direction and *ν* is Poisson’s ratio. Considering this assumption, the X-Y permeability tensor *µ* of isotropic material with the positive saturation magnetostriction *λ_s_* subjected to tensile stresses *σ* in X direction is presented in Figure 1 using polar coordinate parameterization. It should be highlighted that, for such permeability tensor *µ*, the flux density vector *B* in the material is not always perpendicular to the magnetizing field *H*, which is crucial from the point of view of principles of operation of tensductors and pressductors.

As it was proven experimentally, soft magnetic materials are strongly sensitive not only to tensile or compressive stresses, but also to shear stresses [23]. However, it is very sophisticated to assess simultaneous interaction of axial stresses *σ* and shear stresses *τ* on properties of soft magnetic material. To overcome this problem, principal stresses concept was used [24].

**Figure 2:**
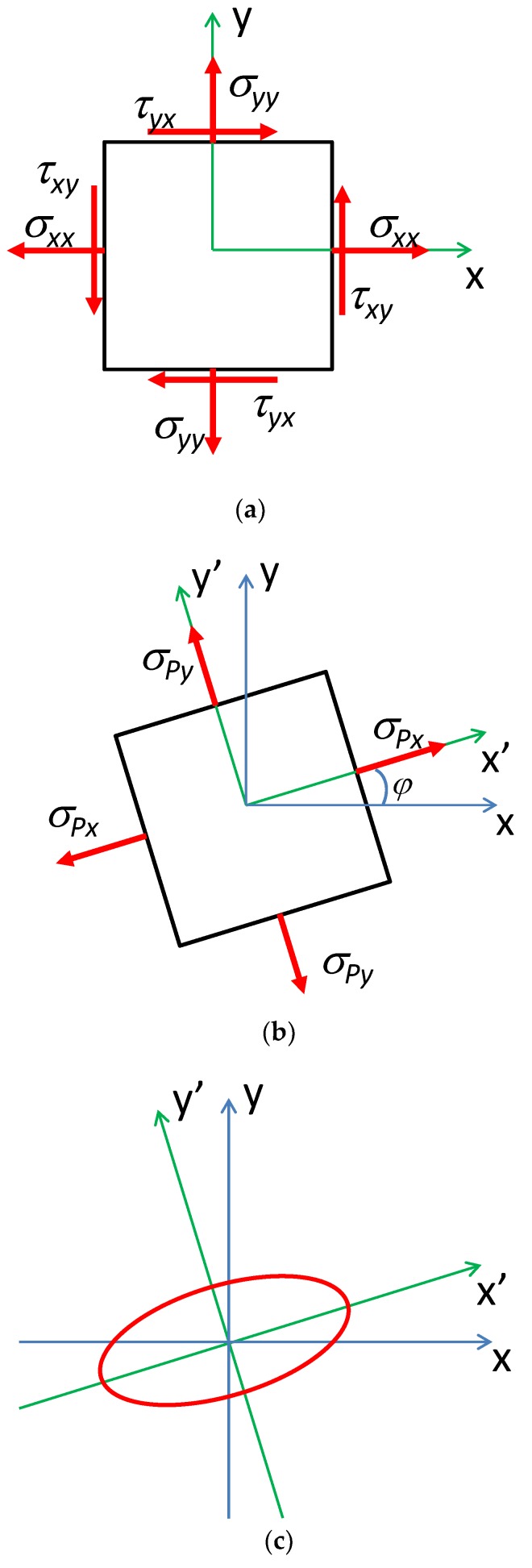
Stress dependence of a permeability tensor *µ* in 2D system: (**a**) a combination of axial and shear stresses *σ* and *τ*, (**b**) principal stresses *σ_P_* representing the same state of stress, (**c**) representation of permeability tensor *µ* depending on the principal stresses *σ_P_*.

As it is presented in Figure 2a,b, the tensile or compressive stress *τ* and shear stress *τ* system can be substituted by the axial stresses with respect to axes that make an angle *φ* with the original axes. These stresses are called principal stresses and enable avoidance of shear stresses *τ* in magnetoelastic analyses. 

It should be highlighted that also the stress dependence of the permeability tensor may be analyzed considering principal stresses. As a result, the permeability tensor (with uniaxial anisotropy) should be rotated in X-Y plane by the angle *φ* determined by the principal stresses. Such a rotated magnetic permeability tensor in a planar coordinate system is presented in Figure 2c.

The magnetic relative permeability tensor *µ_u_* with uniaxial anisotropy with main axes oriented in the direction of X, Y, and Z axes is given as

(2) $$\mu_{u}=\left[\begin{array}{ccc} \mu_{xx} & 0 & 0\\ 0 & \mu_{yy} & 0\\ 0 & 0 & \mu_{zz} \end{array}\right].$$

The stress dependence (in elastic region) of magnetic permeability *µ* of isotropic magnetic material for stresses applied in the same direction as the magnetizing field *H* is given by the *µ(σ)* dependence presented previously in the literature [2,3]. However, for the 2D stress distribution, considering the Equation (1), the effective stresses *σ_xx_* and *σ_yy_* in X and Y direction can be calculated from the following equations:

(3) $$\sigma_{xx}=\sigma_{x}-\nu \cdot \sigma_{y},$$

(4) $$\;\sigma_{yy}=\sigma_{y}-\nu \cdot \sigma_{x}\;.$$

Finally, the parameters of the magnetic permeability tensor in X and Y directions can be calculated as

(5) $$\mu_{xx}=\mu (\sigma_{xx}),$$

(6) $$\;\mu_{yy}=\mu (\sigma_{yy})\;,$$

whereas changes of *µ*_zz_ may be neglected, due to the fact that the sample is magnetized in X and Y directions as a 2D system.

To compensate shear stresses *τ*, the coordinate system for representing the magnetic permeability tensor should first be aligned with the directions of the principal stresses via the rotation by the angle *φ,* so as to replace the axial stresses *σ_x_* and *σ_y_* by the principal stress components *σ_Px_* and *σ_Py_* in the rotated coordinate system. The components of the magnetic permeability tensor can be transformed to the components with respect to the original frame in terms of the rotation matrix *R* [25]:

(7) $$R=\left[\begin{array}{ccc} \cos\left(\varphi \right) & -\sin\left(\varphi \right) & 0\\ \sin\left(\varphi \right) & \cos\left(\varphi \right) & 0\\ 0 & 0 & 1 \end{array}\right].$$

The magnetic permeability tensor *µ_u_* subjected to the influence of principal stresses *σ_Px_* and *σ_Py_* can thus be calculated as

(8) $$\mu_{u}=R\times \left[\begin{array}{ccc} \mu (\sigma_{Pxx}) & 0 & 0\\ 0 & \mu (\sigma_{Pyy}) & 0\\ 0 & 0 & \mu_{zz} \end{array}\right]\times R^{-1},$$

where *σ_Pxx_* and *σ_Pyy_* are calculated according to the Equations (3)–(6).

## 3. Implementation of the Model

**Figure 3:**
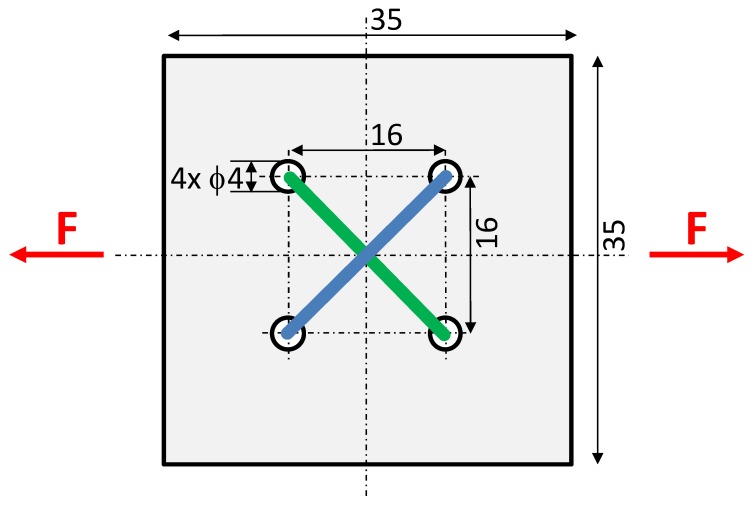
Schematic presentation of the proposed tensductor sensor with a core made of a square-shaped amorphous alloy ribbon; the magnetizing winding (blue), the sensing winding (green).

A tensductor sensor consists of a thin layer of amorphous alloy ribbon core and two perpendicularly oriented coils: a driving coil and a sensing coil [17] as it is presented in Figure 3. The magnetizing coil is connected to a sinewave current source with a frequency of 1 kHz to generate the magnetizing field. Due to the fact that the thickness of the core of the tensductor is 30 µm, the influence of eddy currents may be neglected. For isotropic magnetic material, the flux density *B* component in the direction of the sensing coil is equal to zero. However, when stress-induced axial anisotropy (in x axis direction) appears in the core of the sensor as a result of an external tensile force *F* applied uniformly by special clamps [17], a component of the flux density *B* in the direction of the sensing coil axis appears. As a result, the value of the tensile force *F* can be measured by the measurements of voltage induced on the sensing coil.

The proposed model of the generalization of 2D stress dependence of the magnetic permeability tensor was implemented in MATC language in Elmer FEM open-source environment [26] for finite element modeling. Meshes of tetrahedral elements were created using the NETGEN open-source mesh generator. The Delaunay method of meshing was applied [27]. With the use of Elmer FEM it was possible to estimate mechanical stress distribution in the sensor core and connect it with the magnetic permeability tensor distribution.

**Figure 4:**
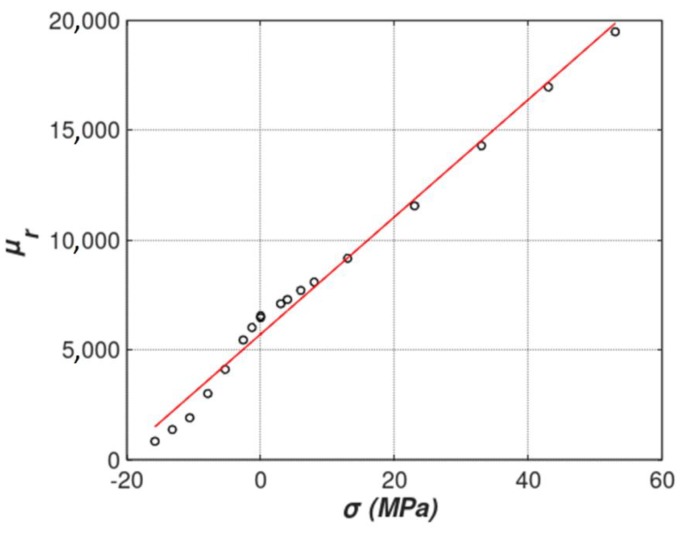
The dependence of relative magnetic permeability *µ_r_* on the axial stress *σ* in a ribbon made of Fe_73.5_Cu_1_Nb_3_Si_15.5_B_7_ amorphous alloy (VITROPERM^®^ 800). Dependence is approximated by linear function (red). Black circles—experimental results.

For the presented modeling linear relative permeability dependence *µ_r_*(*σ*) for axial stresses was proposed. This dependence is presented in Figure 4 and is given by the equation

(9) $$\mu_{r}=5715+267\cdot \sigma \;\left[MPa\right].$$

The proposed dependence was estimated on the base of an experimental test for relatively small values of mechanical stresses in an elastic region of magnetoelastic characteristic. The Fe_73.5_Cu_1_Nb_3_Si_15.5_B_7_ amorphous alloy was used, produced by VACUUMSCHMELZE GmbH & Co. KG (Hanau, Germany). This material was subjected to pre-annealing in the production process and can be treated as nearly-isotropic. Measurements of hysteresis loops of samples magnetized along and across the ribbon length confirm this. The Fe_73.5_Cu_1_Nb_3_Si_15.5_B_7_ amorphous alloy is a precursor of commercially available nanocrystalline VITROPERM^®^ 800 (VACUUMSCHMELZE, Hanau, Germany) [24] and was cast as ribbon 50 mm wide and 30 µm thick. As a result, cross section of the tensductor core near its edge is equal to 1.05 mm^2^. The Young’s modulus for these materials is around 100 GPa, with tensile strength of 1 GPa [28]. In order to measure the magnetoelastic characteristics of this material in the elastic region, two measurement methods were used. In the first of them, the influence of tensile stress along the magnetic induction direction was measured, while in the second the tensile force and induction vectors were perpendicular (based on the method presented in [3]).

**Figure 5:**
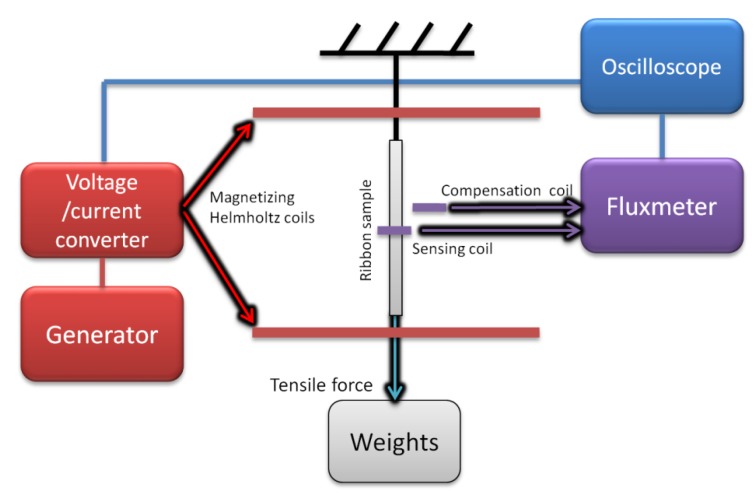
Schematic diagram of the measurement test stand used for the determination of magnetoelastic *B*(*σ*_||_*)* and *µ*(*σ*_||_) characteristics of soft magnetic amorphous ribbon in the tensile stress region.

In the first test stand (Figure 5), the amorphous ribbon sample was placed inside a magnetizing Helmholtz coil, which was connected to the voltage/current converter RDM-2a (WUT, Warsaw, Poland), controlled by the arbitrary function generator (SDG1025, Siglent, Helmond, The Netherlands). The AC current induced the magnetizing field *H*, which in turn produced the magnetic induction *B* in the sample that was measured by the sensing coil placed around the central part of the ribbon. The sensing coil was connected counter-serial with an identical air-flux compensation coil, and to the fluxmeter (model 480, Lakeshore, Westerville, OH, USA). By varying the tensile stress in the sample with suspended weights, the magnetoelastic characteristics of *B*(*σ*_||_) and *µ*(*σ*_||_) were obtained.

**Figure 6:**
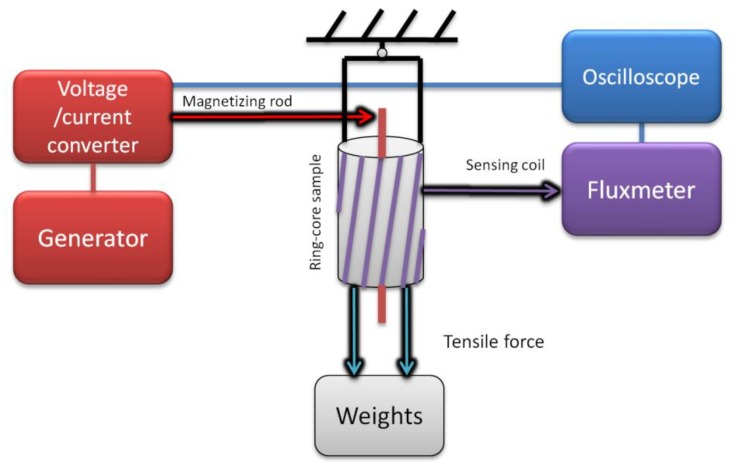
Schematic diagram of the measurement test stand used for the determination of magnetoelastic *B*(*σ*_⊥_) and *µ*(*σ*_⊥_) characteristics soft magnetic amorphous ribbon in the tensile stress region.

The second test stand (Figure 6) employed the same equipment, but the sample was a ring-shaped ribbon-wound core, wound with sensing winding, with one magnetizing rod passing through its center. The application of tensile force along the axis of the ring core produces magnetoelastic characteristics of *B*(*σ*_⊥_) and *µ*(*σ*_⊥_), which, according to [22], after including Poisson’s ratio can substitute *B*∥*σ* magnetoelastic characteristics in the compressive stress region.

In addition, the modeling was carried out by assuming the following mechanical parameters for the tensductor core made of Fe_73.5_Cu_1_Nb_3_Si_15.5_B_7_ amorphous alloy:Young’s modulus: 100 GPa; Poisson’s ratio: 0.3.

It should be also highlighted that due to high Young’s modulus and mechanical hardness in the iron-based amorphous alloys, the elastic region of the stress-strain curve is observed up to the material rupture [29].

## 4. Results of Modeling

**Figure 7:**
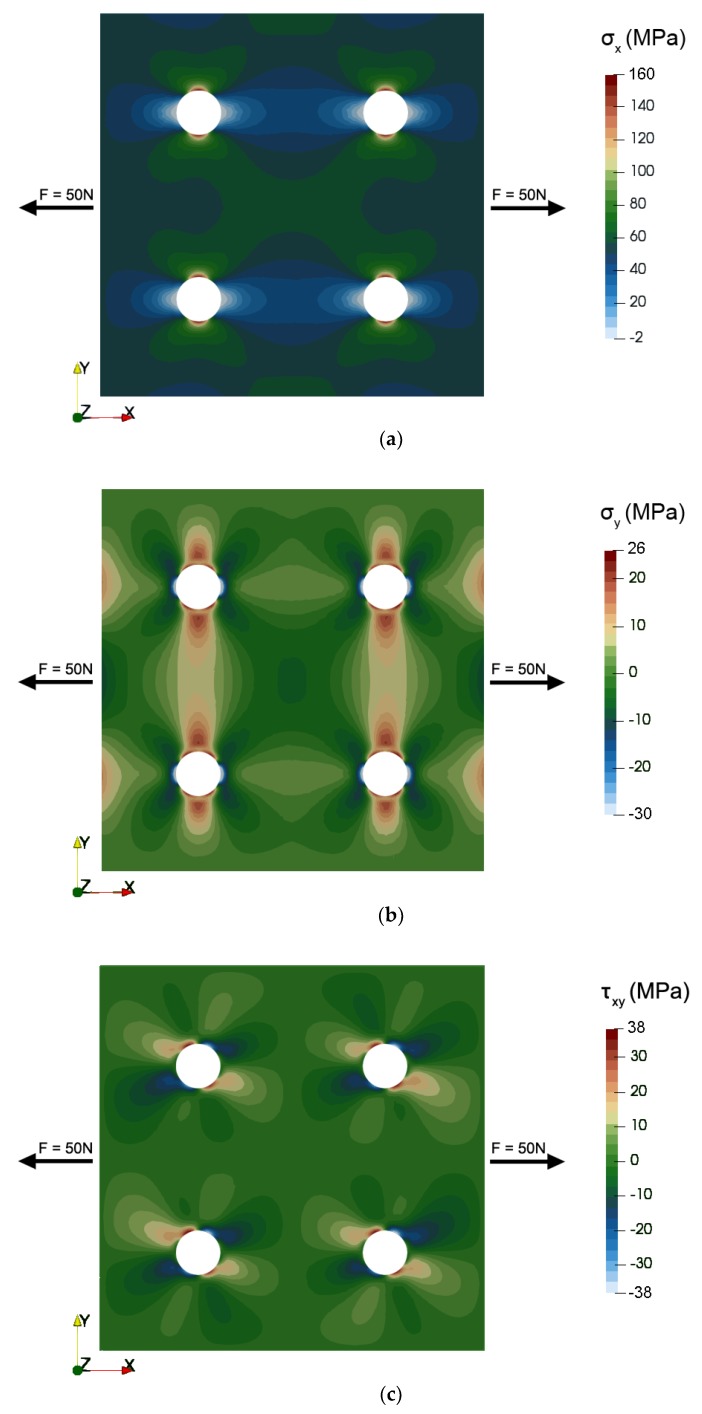
The results of modeling the mechanical stress distribution in the 2D system of tensductor core subjected to tensile force *F* = 50 N in X direction: (**a**) *σ_x_*, (**b**) *σ_y_*, (**c**) *σ_xy_*.

**Figure 8:**
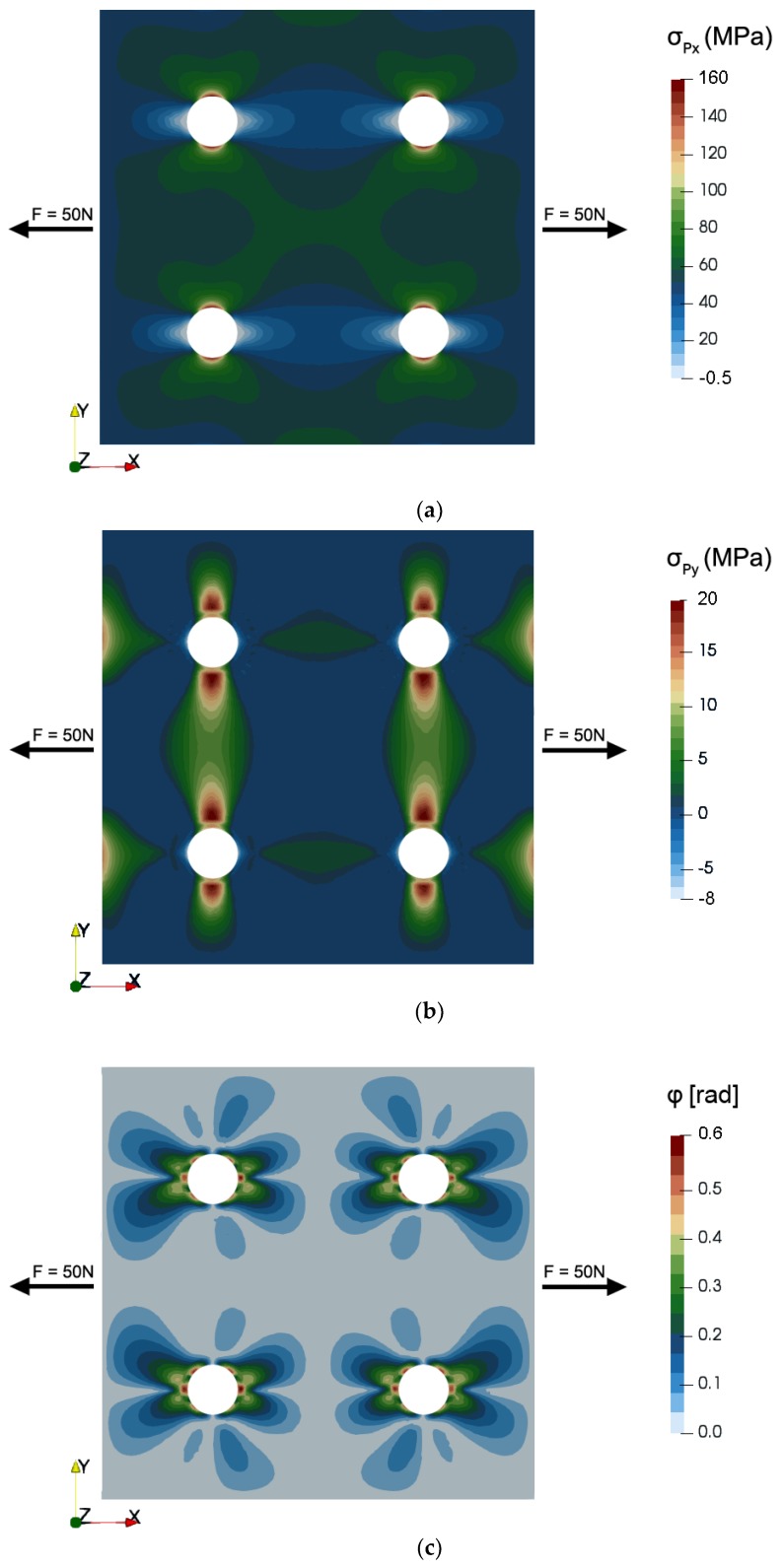
The results of modeling the mechanical stress distribution in terms of principal stresses for the same 2D system of the tensductor core subjected to tensile force *F* = 50 N in X direction: (**a**) *σ_Px_*, (**b**) *σ_Py_*, (**c**) *φ*.

Figure 7 presents the results of modeling the mechanical stress distribution in the 2D system of the tensductor core, showing the axial stresses in X and Y direction as well as the shear stress. Figure 8 presents the same mechanical stress system in terms of the principal stresses in the coordinate system X’-Y’ obtained via rotating by the angle *φ* around the Z axis. It can be observed that the axial stress component corresponding to the first axis dominates in both cases. However, the stress distribution around the holes is sophisticated and may significantly influence the component of flux density *B* responsible for the generation of voltage in the sensing coil.

**Figure 9:**
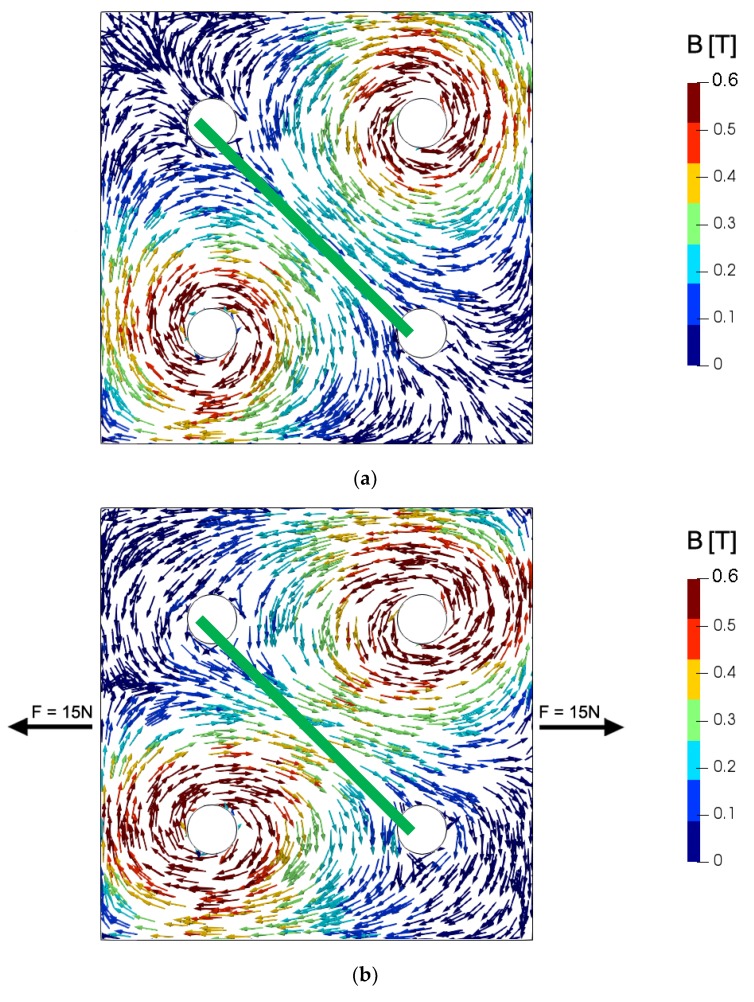
Flux density *B* vector distribution in the core of tensductor sensor: (**a**) without stresses, (**b**) subjected to tensile force *F* = 15 N in X direction. Green line—sensing coil.

Figure 9 presents the flux density *B* vector distribution in the core of the tensductor sensor. It can be seen that the mechanical stresses significantly influence the flux density *B* vector direction by the changes of the magnetic permeability tensor. Moreover, analysis of Figure 9 enables the understanding of principles of operation of the tensductor or pressductor sensor, which is not obvious via consideration of models previously presented in the literature.

**Figure 10:**
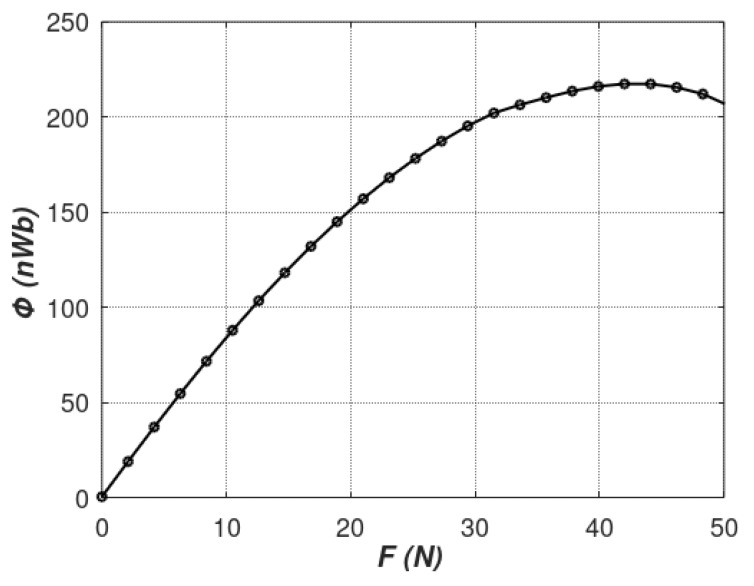
The results of modeling the characteristics of tensductor sensor considering the tensile force *F* dependence of magnetic flux *ϕ* responsible for the generation of voltage in the sensing coil.

Figure 10 presents the results of modeling the characteristics of the tensductor sensor considering the magnetic flux *ϕ* responsible for the generation of the voltage in the sensing coil (the magnetic flux perpendicular to the sensing coil). It should be highlighted that the presented results of modeling are in very good agreement with experimental results presented previously in the literature [17]. As a result, the concept of modeling the stress dependence of the permeability tensor in soft magnetic materials was justified. This will enable the quantitative analyses and optimization of performance of tensductors for industrial applications, as well as understanding and development of other magnetoelastic sensors utilizing the cores made of soft magnetic materials.

## 5. Experimental Verification of the Results

**Figure 11:**
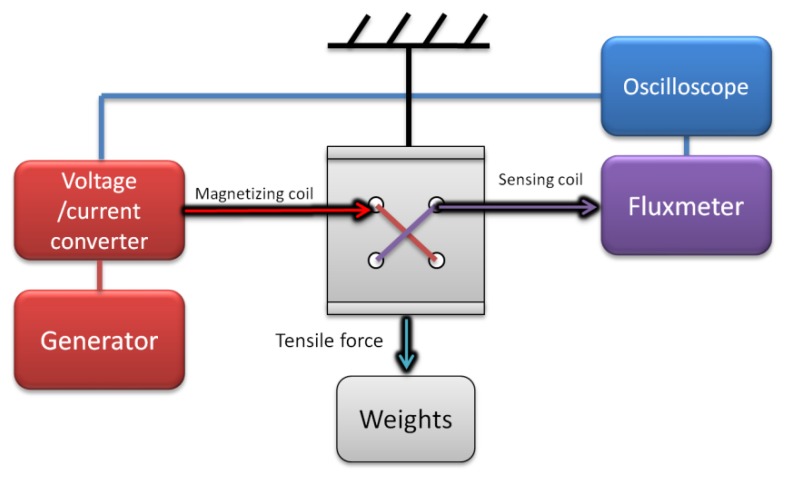
Schematic diagram of tensductor characterization test stand.

In order to compare modeling results with actual tensductor characteristics, the sensor of the same geometry and material was produced and tested on the measurement test stand (Figure 11). Instead of the first harmonic of induced voltage originally proposed for the output of this type of sensor [17], total AC magnetic flux in the sensing coil was measured by means of a sensitive fluxmeter (480, Lakeshore, Westerville, OH, USA).

**Figure 12:**
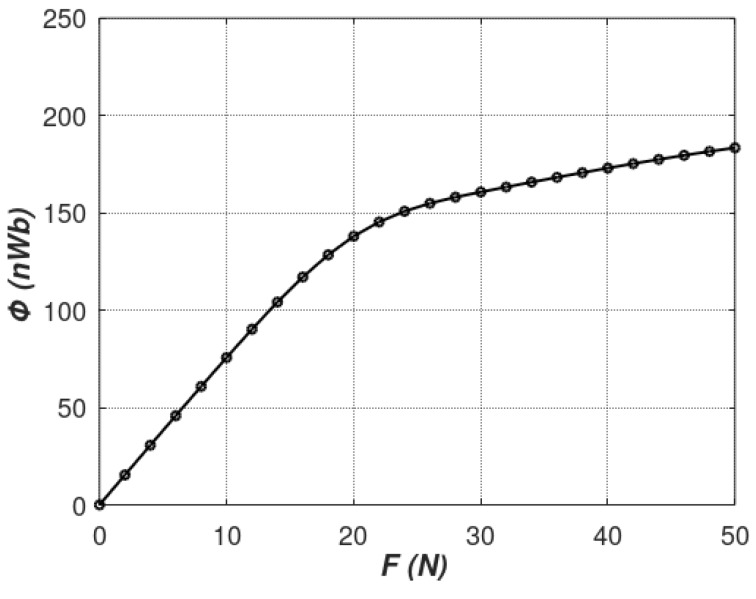
Graph of *B*(*F*)*/ B*(*σ*) characteristics of investigated tensductor tensile force sensor.

The results of the magnetic flux measured by the sensing coil for incrementally raised tensile force are presented in Figure 12.

As can be seen, good correlation with modeling data was achieved for the range 0–20 N of tensile forces. However, for stresses generated by forces higher than 20 N, the linear dependence presented by the Equation (7) seems to be not valid. This phenomenon is connected with the fact that, for higher values of mechanical stresses, the value of saturation magnetostriction of magnetic materials changes significantly [30]. As a result, Villari reversal can be observed [1,2] overruling the linear *µ*(*σ*) dependence. In addition, forces higher than 50 N could not be applied due to the sensor’s mechanical integrity limitations described in [17].

## 6. Conclusions

The paper presents a new concept of modeling the stress dependence of a magnetic permeability tensor and opens new possibilities of modeling the magnetoelastic devices with the finite element method. It should be highlighted that the proposed solution technique may be successfully implemented in both commercial and open-source modeling environments, enabling the tensor description of magnetic properties of soft magnetic materials.

The proposed concept to describe the stress dependence of a magnetic permeability tensor was successfully validated experimentally on the base of measured results of the stress dependence of magnetic properties of Fe_73.5_Cu_1_Nb_3_Si_15.5_B_7_ soft amorphous alloy, which were used for the modeling of tensductor characteristics. These results confirm the correctness of the proposed model for stresses created by tensile forces up to 20 N, which is sufficient from an engineering point of view.

As a result, the proposed concept of modeling the stress dependence of a magnetic permeability tensor can be utilized in the stress-dependent models of inductive components of mechatronics devices. It should be highlighted that the source code for modeling is available under open-source license at www.github.com/romanszewczyk/FEM in the subdirectory “tensductor” for further development and validation.

The main limitation of the method is a lack of magnetic hysteresis reproduction capabilities, which leads to some level of inaccuracy of the method. Future researches will be focused on the implementation of the Preisach [13] or Jiles-Atherton [14] vector-based model of magnetic hysteresis considering the proposed methodology.

## Supplementary Materials

The source code used for modeling is available at www.github.com/romanszewczyk/FEM in the subdirectory “tensductor”. Source code and binaries of ELMER FEM and NETGEN are available at web pages: https://www.csc.fi/web/elmer and https://ngsolve.org/, respectively.
